# Interactive Effects of the Potassium and Nitrogen Relationship on Yield and Quality of Strawberry Grown Under Soilless Conditions

**DOI:** 10.3390/plants9040441

**Published:** 2020-04-02

**Authors:** Pablo Preciado-Rangel, Enrique Troyo-Diéguez, Luis Alonso Valdez-Aguilar, José Luis García-Hernández, José Guadalupe Luna-Ortega

**Affiliations:** 1 National Technologic of Mexico-Torreón Technological Institute (ITT), Torreón, Coahuila 27170, Mexico; 2 Center for Biological Research of Northwest México (CIBNOR S.C.), La Paz, B.C.S. 23096, Mexico; 3 Department of Horticulture, Antonio Narro Agrarian Autonomous University, Saltillo, Coahuila 25315, Mexico; 4 Juárez de Durango State University (UJED)-Agriculture and Zootechnics Faculty (FAZ), Venecia, Durango 34000, Mexico; luis_garher@hotmail.com; 5 Región Laguna Polytechnic University, San Pedro de las Colonias, Coahuila 27948, Mexico

**Keywords:** strawberry, *Fragaria x ananassa* Duch, strawberry yield, fruit quality, nutraceutical quality, N–K nutrition

## Abstract

K and N are the nutrients with the highest influence on yield and fruit quality. From this perspective, the aim of this study was to determine the effect of N as NO_3_^−^, K^+^ and their interactions on the yield and quality of strawberries grown under soilless conditions. A solution comprised of micronutrients based on an amended Steiner’s Universal Nutrient Solution was mixed with 4 levels of K^+^ (5, 7, 9 and 11 mol m^−3^) and 3 levels of NO_3_^−^ (9, 12, and 15 mol m^−3^) to obtain 12 treatments. The results suggest that 15 mol m^−3^ of NO_3_^−^ in the nutrient solution produced the highest yield, but fruit with low nutraceutical quality. On the other hand, 11 mol m^−3^ of K^+^ in the nutrient solution produced the highest yield and fruit with the best nutraceutical quality. The ionic concentration of the Universal Steiner’s Nutrient Solution proved to be the best nutritional option to maximize the yield and nutraceutical quality of strawberry fruit. The increase in NO_3_^−^ concentration in the nutrient solution produced a higher yield of strawberries, while a higher concentration of K^+^ improved fruit quality, thus reaffirming the significance of nutrients within the plant functioning of this crop.

## 1. Introduction

K and N are the most dynamic nutrients, whose deficiency most often affects crop yield and quality, even when only 2 of the 14 essential mineral nutrients for crop nutrition are diagnosed [1]. Of the essential nutrients for strawberry (*Fragaria x ananassa* Duch.) cultivation, N stands out as one of the most important for promoting vigorous plant growth [2], thus allowing for high yields [3]. However, proper precautions should be taken with N-nutrition because, with high concentrations, the nutraceutical quality tends to decrease; in this sense, when there is no N-deficit, the production of N-containing compounds, such as amino acids, proteins and alkaloids, is privileged [4,5]. On the other hand, K^+^ increases phenolic composition and antioxidant capacity [6]; it also plays a role in enzymatic activation, protein synthesis and photosynthesis [7]. The relationship between N and K determines the balance between vegetative growth, fruit quality and reproductive processes. K plays the role of a growth regulator when the availability of N is high, also ensuring adequate fruit formation. K also regulates the appearance of certain physiological disorders that affect the internal and external appearance of fruit, mainly color, and constitutes an aspect of agronomic management related to the fruit’s durability [8,9,10].

On the other hand, the shortage of water in arid zones is exacerbating the need to adopt new horticultural crops, such as strawberries, which efficiently use water. Strawberries can be considered a highly productive option and can, additionally, be of great value in diversifying food and improving nutrition in arid areas. Strawberries are a rich source of a wide variety of nutritive compounds, such as sugars, vitamins and minerals, as well as non-nutritive, bioactive compounds, such as flavonoids, anthocyanins and phenolic acids. All of these compounds exert a synergistic and cumulative effect on human health promotion and in disease prevention [11].

The aim of this study was to determine the effects of N as NO_3_^−^, K^+^ and their interactions on the yield and quality of strawberries grown under soilless conditions.

## 2. Results

### 2.1. Plant Variables

After data capture and analysis, and because 4 K levels did not influence numerical trends, the data obtained from the 12 original treatments were re-examined by independent analysis of variance. The highest yield, number of fruit and firmness were obtained with treatments of 12 and 15 mol m^−3^ of NO_3_^−^-N in the nutrient solution (Table 1); nevertheless, with 9 mol m^−3^, a larger fruit size was achieved; the latter refers to the equatorial and polar diameter, average weight of fruit and ^o^ Brix. It should be noted that with 12 mol m^−3^ of NO_3_^−^-N, intermediate values were registered between 9 and 15 mol m^−3^ for the aforementioned variables. Data for yield, fruit size, fruit weight, and the results for ^o^ Brix and firmness were statistically similar among treatments with 12 and 15 mol m^−3^ of NO_3_^−^-N with doses of 9 and 11 mol m^−3^ of K^+^, except for the variable number of fruit. Specifically, the optimum concentration of NO_3_^−^-N to achieve significant yields was 12 mol m^−3^ with 11 mol m^−3^ of K^+^ in the nutrient solution. It is known that one of the main components of yield is the number of fruit, although other physiological attributes are also involved [12]. However, yield is regulated mainly by the N supplement [13], which was confirmed by our results to participate directly in the synthesis of amino acids, proteins and nucleic acids and also improve weight and size of fruit and favor yield; K^+^ improves the fruit’s quality [2,14].

The highest accumulation of total soluble solids (° Brix) in strawberries corresponded to the plants treated with the nutritive solution with 9 mol m^−3^ of NO_3_^−^ and 11 mol m^−3^ of K^+^. However, it was observed that as the concentration of NO_3_^−^ (12 and 15 mol m^−3^) in the nutritional solution increased, the organoleptic quality of strawberries decreased along with the accumulation of sugars in the fruit [15]. This effect is a consequence of an adequate supply of water and N to the crop through irrigation [16], which dilutes the accumulation of soluble solids into the fruit. Similar results were obtained by Andriolo et al. [17], who found a decrease in soluble solids in treatments with high doses of nitrogen. Regarding the nutrient factor K^+^, an increase in the average weight of fruit, and consequently in yield, when high concentrations of K^+^ were used was reported elsewhere [18,19]; in this sense, results showed the relevant role of K^+^ as a main nutrient for an adequate yield [7,20]. The results suggested the existence of a relationship between yield and K^+^. The refractometer index (soluble solids) is considered to be one of the most important fruit quality criteria [21]. According to the results of the analysis of variance, the highest accumulation of soluble solids in the irrigated fruit was obtained with the highest dose of K^+^ (11 mol m^−3^) with 10.6 ° Brix. This result agrees with Gallace and Lieten [22], who obtained the best results in ° Brix with increases in K^+^; in this sense, K^+^ has a preponderant function within the plant and in the transport of solutes through the phloem, including the movement of sugars to the fruit [21,23].

### 2.2. Total Antioxidants

The phytonutrients content is a relevant parameter related to the quality of fruit, and is mainly determined by genotype and fertilization [15]. The antioxidant capacity of fruit and vegetables raises interest in the prevention and control of certain human diseases, such as cancer [24]. The total antioxidant content in fruit showed significant differences between N levels (p ≤ 0.05), reaching the highest value with 9 mol m^−^^3^ of K^+^. The highest values were detected the in fruit of the plants irrigated with the nutritive solution of 12 mol m^−3^ of NO_3_^−^ with 6304.6 mequivTrolox/100 g of fresh base (BF); this level of NO_3_^−^ seems to be optimal with 9 mol m^−^^3^ of K^+^ in the nutritive solution for the increase of the antioxidant capacity in fruit. At a higher dose of NO_3_^-^, the quality of the fruit was negatively affected. Phenolic compounds are important to the taste of strawberries; the sugar/acid ratio is used to determine the optimum harvest time because it is a precise quality index [25]. According to the ANOVA for this variable, it was found that by increasing the K^+^ levels from 5 to 7 mol m^−3^, the highest values for the phenolic content in fruit were reached; however, for the antioxidant capacity, the best dose of K^+^ was 9 mol m^−3^. This dose represents the optimum amount of K^+^ because of its function as an activator of physiological reactions and its important role in growth and efficiency of water use [26]. This result contrasts with the concentrations of 9 and 11 mol m^−3^ of K^+^, at which the phenolic content decreased (Table 2).

### 2.3. N–K Interaction

The NO_3_^−^–K^+^ interaction showed no significant difference for equatorial diameter, polar diameter, average fruit weight, total soluble solids and firmness, while for the number of fruit significant differences were recorded (p ≤ 0.05;). It was found that the concentration of nitrates in the nutrient solution was positively associated with fruit per plant; in this case, with 12 and 15 mol m^−3^ of NO_3_^−^, the best interaction with K^+^ was at 7 mol m^−3^ of K^+^. Our results coincided with those of Ebrahimi et al. [27], who reported that increasing the levels of N and K in the nutrient solution increased the number of fruit per plant. Accordingly, in other studies, doses of 7.6 meq L^−1^ of K^+^ and 14.2 mol m^−3^ of NO_3_^−^ in the nutritive solution increased the production of strawberries [28,29]. The analysis of variance (p ≤ 0.05) for the effects of N and K evidenced that the ion ratios recommended by the Steiner solution [30] ensured the highest yields. Their amounts were (mol m^−3^), 60/5/35 for NO_3_^−^/H2PO_4_^−^/SO_4_^2^^−^ and 35/45/20 for K^+^/Ca^2+^/Mg^2+^.

### 2.4. Phenolic Compounds

It has been postulated that relative differences in nutrient release from various fertilizers could lead to different C/N and N/K ratios and, consequently, to differences in the production of secondary metabolites, such as phenolic compounds, in plants [1,31]. According to the analysis of variance (p ≤ 0.05), the best results were obtained with the ratio 12 mol m^−3^ of NO_3_^−^ and 7 mol m^−3^ of K^+^. This concurs with previous reports [32,33] that an excessive increase in N fertilization reduces the accumulation of phenolic compounds, which are highly relevant to the quality of fruit. There is a particular interest in understanding the potential effects of nutrients in metabolic and physiological processes.

### 2.5. Total Antioxidants

Potassium stands out as the cation that has the greatest influence on the quality parameters that determine the commercialization of fruit, consumer preferences and the concentration of associated phytonutrients of vital importance to human health [34]. In relation to this variable, the present study found favorable results in terms of total antioxidant capacity in fruit; the best N–K interaction was detected with 12 mol m^−3^ NO_3_^−^ and 5 mol m^−3^ K^+^ in the nutritive solution to give the optimum antioxidant capacity in strawberries.

## 3. Discussion

It is proven that plants produce higher amounts of primary (simple and complex sugars) and secondary (terpenoids, phenolic compounds, pigments, vitamins and organic acids) metabolites when N availability is optimal [35,36]. N and K, as well as their interaction, affected the yield and quality of strawberries, but the application of different concentrations of K did not show significant effects on the antioxidant capacity of strawberries.

Macronutrients play a very important role in plant growth and development. Their functions range from being structural units to being redox-sensitive agents. In general, application of macronutrients increases the yield, growth and quality of crops [37]. Similar studies focused on N and K interactions in different crops, including maize [38] and pepper [39]; these works provided important corrections to the K/N ratio. In another study of pepper, a misbalance in the K/N ratio affected negatively the metabolism of carotenoids, thus decreasing the antioxidant capacity of fruit [40]. In this context, the importance of a balanced K/N ratio was observed in wild blueberries by Percival and Sanderson [41]. In another study performed on Cabernet Sauvignon grape, increasing ratios of N/K caused an increment in the phenolic content per gram of fruit during a period of two years [42].

In the present study, the results can be attributed to the fact that strawberry itself is a natural source of antioxidants and related compounds. It was observed that by increasing fertilization from 12 to 15 mol m^−3^ of NO_3_^−^, the antioxidant capacity decreased. In this sense, it can be established that the absence of a nitrogen deficiency is associated with a decrease in the nutraceutical quality, because the production of N-containing compounds, such as amino acids, proteins and alkaloids, is privileged [5,43].

Managing different levels of application of N–K nutrients and their interaction in the nutrient solution allows us to prepare a nutrient solution with optimal concentrations of nutrients, according to the prioritization of parameters in strawberry.

## 4. Materials and Methods

### 4.1. Study Site and Experimental Conditions

**Figure 1:**
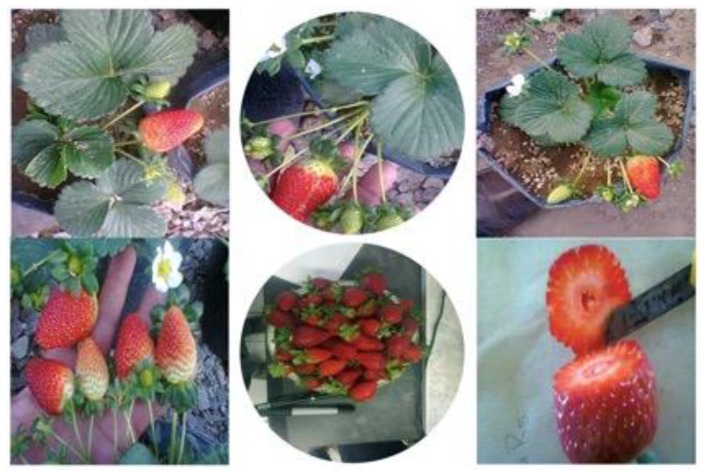
Strawberry grown under soilless conditions with the effects of N/K interaction, northern Mexico.

This study was carried out between September 2017 and February 2018 in the tunnel greenhouse of the Technological Institute of Torreón (ITT), located 7.5 km from the Torreón-San Pedro highway, in the municipality of Torreón, Coahuila, Mexico (N 25°36’36.54”; W 103°22’32.28”), at an altitude of 1123 m. Torreón is part of the region known as “Comarca Lagunera” or “La Laguna.” The climate of Comarca Lagunera is of a desert type with low atmospheric humidity and an average annual rainfall of 240 mm; the rainy period covers the period from May to September, with 70% of the precipitation falling during this interval. In most of the region, there is an annual evaporation of 2600 mm and an average temperature of 20 °C [44]. In the environmental context, the short period of availability of water motivates the development of new horticultural crops with efficient technologies in the use of water. Inside the greenhouse, the daytime temperature averaged 29.5 °C, while the night temperature was 18.4 °C. Strawberry plants (*Fragaria x ananassa* Duch.) were grown in a substrate based on perlite/sand (80/20, v/v) previously sterilized, in polyethylene black bags weighing 10 L; plastic bags were placed as pots in a double row with a density of 9 plants per m^2^, above the soil, establishing 1 plant per pot (Figure 1).

### 4.2. Treatments and Experimental Design

The evaluation included three concentrations of N as NO_3_^−^ (9, 12 and 15 mol m^−3^) and four concentrations of K^+^ (5, 7, 9 and 11 mol m^−3^). Nutrients NO_3_^−^ and K^+^ were obtained from potassium nitrate fertilizer (KNO_3_), made with formulation 12-0-46 (NPK), 96% purity and a solubility of 315 g L^−1^, with a pH of 7. KNO_3_ is a compound fertilizer that contains N in nitric form, which takes immediate effect, and K, which has a delayed effect. Treatments were designed based on a modification of Steiner’s Universal Nutrient Solution (1961) [30], which was added to the 3 NO_3_^−^ and 4 K^+^ concentrations, resulting in 12 nutrient solutions (Table 3). The solutions were complemented with a mixture of micronutrients, with the following concentrations (mg·L^−1^): 1.6 of Mn, 0.11 of Cu, 0.865 of B, 0.023 of Zn, 0.048 of Mo and 5 of Fe. The resulting solutions were adjusted to an osmotic potential of −0.073 MPa and a pH of 5.5. In each case, the solution was applied at a dose of 960 mL–1 L per plant per day, through a drip irrigation system programmed with a timer to supply 8 irrigations of 2 minutes each, allowing a light drainage of around 20% to maintain a humidity level at field capacity. A completely randomized design was used, considering 10 repetitions per treatment, taking a pot as an experimental unit.

### 4.3. Fruit Yield and Quality

The yield was recorded by weighing the fruits of each plant. The fruit size was recorded by measuring the polar and equatorial diameter using a digital caliper. For fruits, the refractometric index in ° Brix was determined with a manual refractometer from 0% to 32% (Atago^®^ Master 2311). The firmness of the fruit was determined with an Extech penetrometer (FHT200), using a 5 mm diameter plunger on the opposite sides of the fruit, taking an average of two measurements in Newton.

### 4.4. Determination of Nutraceutical Quality

The nutraceutical quality was determined using the following procedure:

(a) Obtaining extracts. Samples of 5 g of fresh-ground strawberry pulp were mixed with 10 mL of methanol in plastic tubes with screw caps, then placed on a rotary shaker (ATR Inc) for 6 h at 20 rpm at 5 °C. The tubes with the mixture were then centrifuged at 3000 rpm for 10 min and the supernatant was removed for analysis.

(b) Total phenolic content (TPC). TPC was measured by a modification of the Folin–Ciocalteu method [45]. A sample of 30 µL was mixed with 270 µL of distilled water in a test tube and 1.5 mL of diluted Folin–Ciocalteu reagent (Sigma Aldrich) (1/15) was added, vortexing for 10 s. After 5 min, 1.2 mL of sodium carbonate (7.5% w/v) was added while stirring for 10 s. The solution was placed in a water bath at 45 °C for 15 min and then allowed to cool to room temperature. The absorbance of the solution was read at 765 nm on a 4000 spectrophotometer (Hach^®^, Ames, Iowa, USA). The phenolic content was calculated by a reference curve using gallic acid (Sigma Aldrich) as a standard; the results are reported in mg of equivalent gallic acid per g of base sample (fresh mg equiv AG/g BF).

(c) Equivalent antioxidant capacity in Trolox (6-hydroxy-2,5,7,8-tetramethylchroman-2-carboxylic acid, a synthetic derivative of vitamin E, Aldrich) (method DPPH^•^ (2,2-diphenyl-1-picrylhydrazyl, Aldrich) or DPPH radical that is reduced by fruit antioxidants). The antioxidant capacity was evaluated according to the DPPH^•^ in vitro method, using a modification of the method published by Brand-Williams [46]. A solution of DPPH^•^ was prepared in methanol, adjusting the absorbance to 515 nm at 1.100 ± 0.01. For the determination of the antioxidant capacity, a sample of 50 µL was mixed with 950 µL of DPPH^•^ solution; after 3 min of reaction, the absorbance of the mixture was read at 515 nm. A standard curve was prepared with Trolox and the results are reported as equivalent antioxidant capacity in µM equivalent in Trolox per g fresh base (µM equiv Trolox/g BF).

(d) Equivalent antioxidant capacity (EAC) in Trolox—ABTS cation radical method or ABTS^•+^ (2,2’-azino-bis(3-ethylbenzothiazoline-6-sulfonic acid), Aldrich). The EAC in Trolox was evaluated according to the ABTS^•+^ method [45,47]. A solution with 40 mg of ABTS and 1.5 g of manganese dioxide (Fermont) was prepared in 15 mL of distilled water. The mixture was stirred vigorously and allowed to stand while covered for 20 min to form the green-colored ABTS^•+^. It was then filtered on Whatman 40 paper (GE Healthcare) and the absorbance at 734 nm was adjusted to 0.700 ± 0.010 using a 5 Mm phosphate buffer solution. For the determination of antioxidant capacity, a sample of 100 µL was added with 1 mL of ABTS^•+^. After 60 and 90 s of reaction, the absorbance was read at 734 nm. A standard curve with Trolox (Aldrich) was prepared. The results are reported as the equivalent of antioxidant capacity in µM equivalent in Trolox per g of fresh base (µM equiv Trolox/g BF).

### 4.5. Statistical Analysis

The data on the response variables for the factors under study, as well as their interactions (N–K), were analyzed by means of a variance analysis using the Statistical Analysis System statistical software version 9.1 (SAS Institute, USA) [48]. Tukey’s test was used (p < 0.05) to compare means.

## 5. Conclusions

The optimal concentration of N to maximize yield and number of fruits was not reached under the experimental conditions of this study, since there was a linear trend on these study parameters. We expect to obtain better results by increasing the concentrations of N in the nutrient solution. With a concentration of 9 mol m^−3^ of NO_3_^−^ in the nutritive solution, the highest accumulation of total soluble solids in strawberries was obtained, while with 12 mol m^−3^ of NO_3_^−^ in the solution, more fruit were produced per plant; yield and antioxidant capacity in strawberries increased as well. A concentration of 11 mol m^−3^ of K in the nutrient solution resulted in the greatest accumulations of phenolic compounds in strawberries. With high concentrations of K (11 mol m^−3^), the soluble solids and yield were significantly improved. In the N–K interaction, a concentration of 7 mol m^−3^ of K^+^ and 12 mol m^−3^ of N maximized the yield, antioxidant capacity and phenolic compounds.

## Figures and Tables

**Table 1:** Mean values for yield (YIELD), number of fruit (NF), equatorial and polar diameter (DE and DP, respectively), fruit weight (AFW), soluble solids (° Brix) and Firmness (FIRM) for the levels and interactions of N and K in the nutrient solution in strawberries under soilless conditions.

FACTOR	Conc	YIELD	NF	DE	DP	AFW	° BRIX	FIRM
	mol m^−3^	g	- - - - - - - - mm - - - - - - - -			g		Newton
	9	89.3 b	7.03 b	27.4	36.9	13.4	10.5 a^§^	2.99
NO_3_^−^	12	108 a	8.08 b	26.9	36.3	13.3	10.0 ab	3.34
	15	111 a	9.37 a	26.2	34.6	12.2	9.51 b	3.56
	5	90.8 b	7.94	26.9	36.2	13.1	9.30 b	3.04
K^+^	7	102 ab	8.55	26.6	35.0	12.5	9.69 b	3.56
	9	103 ab	7.44	27.1	35.5	12.5	9.73 ab	3.38
	11	114 a	8.66	26.9	37.1	13.6	10.6 a	3.31
N*K		*	*	ns	ns	ns	ns	ns

Values with equal letters within each column and factor are equal according to Tukey’s test (p ≤ 0.05). * = significant; ns = non-significant according to Tukey’s test (p ≤ 0.05).

**Table 2:** Antioxidant capacity and phenolic compounds in strawberry fruit with different levels and interactions of N and K in the nutrient solution.

Factor	Levels mol m^−3^	Antioxidant Capacity meqTrolox/100 g BF	Phenolic Compounds mg equiv AG/g BF
	9	6090 ab^§^	1078
NO_3_^−^	12	6305 a	1081
	15	5650 b	1065
	5	6103	1073 ab
K^+^	7	5822	1130 a
	9	6140	994 b
	11	5996	1102 ab
N*K		*	*

^§^ Values with the same letters within each column are statistically similar (Tukey; p ≤ 0.05). *: significant; ns: non-significant according to Tukey’s test (p ≤ 0.05).

**Table 3:** Nutritional composition of the solution with the treatments applied in the cultivation of strawberries under soilless conditions.

Treatments	NO_3_^−^	H_2_PO_4_^−^	S0_4_^2^^−^	K^+^	Ca^2+^	Mg^2+^
	--------------------------------------- mol m^−3^ -----------------------------------------					
1 (9 NO_3_^−^, 5 K^+^)	9.75	1.49	10.4	5.41	11.2	4.99
2 (9 NO_3_^−^, 7 K^+^)	9.41	1.43	10.1	7.32	9.41	4.18
3 (9 NO_3_^−^, 9 K^+^)	9.09	1.38	9.72	9.09	7.69	3.41
4 (9 NO_3_^−^, 11 K^+^)	8.79	1.34	9.41	10.8	6.09	2.69
5 (12 NO_3_^−^, 5 K^+^)	12.4	1.03	7.24	5.17	10.7	4.76
6 (12 NO_3_^−^, 7 K^+^)	12.0	1.00	7.00	7.00	9.00	4.00
7 (12 NO_3_^−^, 9 K^+^)	11.6	0.96	6.77	8.71	7.36	3.27
8 (12 NO_3_^−^, 11 K^+^)	11.3	0.93	6.56	10.3	5.84	2.58
9 (15 NO_3_^−^, 5 K^+^)	14.8	0.61	4.33	4.94	10.3	4.56
10 (15 NO_3_^−^, 7 K^+^)	14.4	0.59	4.19	6.70	8.62	3.83
11 (15 NO_3_^−^, 9 K^+^)	13.9	0.58	4.06	8.35	7.06	3.13
12 (15 NO_3_^−^, 11 K^+^)	13.5	0.56	3.94	9.90	5.61	2.48
